# Editorial

**Published:** 1896-11

**Authors:** 


### Editorial.

### CRATÆGUS OXYACANTHA IN THE TREATMENT OF HEART DISEASE.

In a recent communication to the *New York Medical Journal*, Dr. M. C. Jennings, of Chicago, calls attention of the profession to a new or rather lately announced cure for certain forms of heart disease. The remedy was used by an Irish charlatan who died about two years ago, and the secret of it was not divulged until after his death, when it was announced as consisting of a fluid extract of cratægus oxyacantha or hawthorn fruit.

The author prepared a fluid extract according to the British pharmacopeia, and has used it on forty-three patients suffering from various forms of heart disease, organic and functional, with very gratifying results; all having been apparently cured. The author wisely expresses misgivings of actual cure in cases of organic lesion.

The physiological action of the drug appears to be an inhibitory effect upon the vagi and cardiac centres, diminishing the pulse rate, increasing intra-ventricular pressure, and filling the heart with blood causes retardation of the beat, and establishes an equilibrium between the general blood pressure and the force of the beat.

Under its use the cardiac impulse is strengthened and regulated, and the central nervous system is favorably influenced. The appetite increases, assimilation and nutrition improve, showing an influence over the sympathetic and solar plexus. There is also marked improvement in the mental stability of patients taking the drug. Fatty degeneration of the heart is a contra-indication to its use.

The dose of the fluid extract is ten to fifteen drops after meals—taken before food, it is liable to produce nausea.

The treatment usually extends over three months, discontinuing for a week or so after the first month.

Supportive and tonic treatment is indicated in conjunction with the use of cratægus, and digitalis is a useful adjunct.

We can find no reference to this drug in the several materiæ medicæ consulted. Foster's Dictionary defines cratægus as a Linnean genus of rosaceous plants of the Pomeæ, and gives twenty-one species, including the oxyacantha of Dr. Jennings, which is also called *mespilus oxyacantha*. There is no suggestion in this reference to the therapeutic use of the plant.

*C. parvifolia*, the common hawthorn, grows abundantly in the Southern States, and may be used instead of the oxyacantha, by those who desire to test the assertions of the *New York Medical Journal's* correspondent. It seems to merit a trial.

---

### SOUTHERN SURGICAL AND GYNECOLOGICAL ASSOCIATION.

The ninth annual meeting of the Association will be held in Nashville, Tenn., Tuesday, Wednesday and Thursday, November 10th, 11th and 12th, 1896. The Nicholson House has been selected as headquarters for the Association. The rates will range from $2.00 to $4.00 per day. Arrangements have been made with the Southern Passenger Association for reduced rates on the certificate plan. These certificates, properly signed, will entitle those who attend to a return ticket at the rate of one-third of one full fare. Those who contemplate attending the Pan-American Medical Congress, to be held in the city of Mexico, November 16–19, will have time to do so after the meeting of the Southern Surgical and Gynecological Association. A rate of one fare for the round trip has been made on account of the Congress, stop-over privileges being allowed holders of tickets therefor.

The following is a partial list of the papers to be read:

President's address, E. S. Lewis, M. D., New Orleans, La.

Memorial address on Dr. Paul F. Eve, Richard Douglas, M. D., Nashville, Tenn.

Acute mania following surgical operations, Joseph Price, M. D., Philadelphia, Pa.

Cholelithiasis, with report of cases, A. M. Cartledge, M. D., Louisville, Ky.

Treatment of carcinoma uteri, Jas. T. Jelks, M. D., Hot Springs, Ark.

Maternal impressions and their influence upon the fœtus in utero; illustrated by photographs; C. H. Mastin, M. D., Mobile, Ala.

Ureteral anastomosis, J. W. Bovee, M. D., Washington, D. C.

Compound comminuted fracture of the radius and ulnar, near the wrist joint, H. M. Hunter, M. D., Union Springs, Ala.

Enchondroma of the pelvis, with report of a successful operation, Rudolph Matas, M. D., New Orleans, La.

The treatment of pregnancy and labor complicated by fibroid tumors of the uterus, Henry D. Fry, M. D., Washington, D. C.

Uterine drainage as a factor in the prevention and relief of pelvic inflammation, R. R. Kime, M. D., Atlanta, Ga.

Splitting the capsule for nephralgia, with report of cases, George Ben Johnston, M. D., Richmond, Va.

Gunshot wounds of the abdomen, W. E. Parker, M. D., New Orleans, La.

Vaginal drainage in surgery of the uterus and other pelvic structures, W. H. Wathen, M. D., Louisville, Ky.

Mental complications following surgical operations, J. T. Wilson, M. D., Sherman, Texas.

Tracheotomy versus intubation, Chalmers Deaderick, M. D., Knoxville, Tenn.

The treatment of inevitable abortion, Virgil O. Hardon, M. D., Atlanta, Ga.

Some remarks on fractures, W. F. Westmoreland, M. D., Atlanta, Ga.

Hypodermoclysis in severe railway injuries requiring multiple operations, James Evans, M. D., Florence, S. C.

Wounds of the liver, with report of cases, S. M. Fortier, M. D., New Orleans, La.

Peculiarities of the surgical diseases and injuries of the neck, Edmond Souchon, M. D., New Orleans, La.

Clamps instead of ligatures in vaginal hysterectomy, H. S. Cocram, M. D., New Orleans, La.

Contributions to the pathology of adherent placenta, Bedford Brown, M. D., Alexandria, Va.

The dry method in intra-uterine surgery, Edwin Walker, M. D., Evansville, Ind.

Subject to be announced, J. D. S. Davis, M. D., Birmingham, Ala.

Subject to be announced, L. S. McMurtry, M. D., Louisville, Ky.

Subject to be announced, W. B. Rogers, M. D., Memphis, Tenn.

Subject to be announced, Geo. H. Noble, Atlanta, Ga.

Subject to be announced, N. P. Dandridge, M. D., Cincinnati, Ohio.

Subject to be announced, Geo. J. Engelmann, M. D., St. Louis, Mo.

Subject to be announced, Jas. McF. Gaston, M. D., Atlanta, Ga.

Subject to be announced, Cornelius Kollock, M. D., Cheraw, S. C.

An experimental study of bile in the peritoneal cavity, W. E. B. Davis, M. D., Birmingham, Ala.

Discussion on appendicitis.

Discussion on vaginal versus abdominal section for pus in the pelvis.

Members of the medical profession are cordially invited to attend.

Dr. W. D. Haggard, of Nashville, is chairman of the committee of arrangements.

E. S. Lewis, M. D., President.

W. E. B. Davis, M. D., Secretary.

